# Comparison of Antiviral Immune Responses in Healthy Cats Induced by Two Immune Therapeutics

**DOI:** 10.3390/pathogens13070602

**Published:** 2024-07-22

**Authors:** Petra Cerna, Steven Dow, William Wheat, Lyndah Chow, Jennifer Hawley, Michael R. Lappin

**Affiliations:** Department of Clinical Sciences, Colorado State University, Fort Collins, CO 80523, USA; william.wheat@colostate.edu (W.W.); jennifer.hawley@colostate.edu (J.H.)

**Keywords:** FCoV, coronavirus, FIP, innate immune response, cytokines, T cell

## Abstract

Background: Effective immunotherapeutic agents for use in cats are needed to aid in the management of intractable viral diseases, including feline infectious peritonitis (FIP) infection. The objectives of this study were to compare two different immune stimulants for antiviral activity in cats: (1) TLR 2/6-activating compound polyprenyl immunostimulant; (PI) and (2) liposome Toll-like receptor 3/9 agonist complexes (LTCs) to determine relative abilities to stimulate the induction of type I (IFN-α, IFN-β) and type II (IFN-γ) interferon immune responses in vitro and to study the effects of treatment on immune responses in healthy cats. Methods: Cytokine and cellular immune responses to PI and LTC were evaluated using peripheral blood mononuclear cells (PBMCs) from healthy cats incubated with LTC and PI at indicated concentrations using reverse transcriptase polymerase chain reaction assays and ELISA assays. The effects of the immune stimulants on inhibiting FIPV replication were assessed using a feline macrophage cell line (fcwf-4). Cytokine and cellular immune responses to PI and LTC were evaluated in blood samples from healthy cats treated with PI and LTC, using reverse transcriptase polymerase chain reaction (RT-PCR) and ELISA assays. Results: In the in vitro studies, both compounds triggered the upregulated expression of IFN-α, IFN-γ, and IL-1β genes in cat PBMC, whereas treatment with LTC induced significantly greater expression of IFN-α and IFN-γ on Day 1 and IL-1b on Day 3. There was significant protection from FIPV-induced cytopathic effects when fcwf-4 cells were treated with conditioned medium from LTC-activated leukocytes. In the healthy cat study (in vivo), both PI and LTC increased the mRNA signal for IFN-α, IFN-γ, and IL-1β above baseline at multiple time points with statistically greater increases in the LTC group on either Day 1 (IFN-α, IFN-γ) or Day 3 (IL-1β). In addition, RANTES increased over time in cats treated with the LTC. Conclusions: Both LTC and PI protocols induced immune-enhancing effects, suggesting a possible clinical use for the management of chronic infectious diseases like FIP. Activating the TLR 3 and 9 pathways (LTC) induced superior broad interferon production in vitro than the activation of the TLR 2 and 6 pathways (PI).

## 1. Introduction

Feline infectious peritonitis (FIP) has been a challenge for veterinarians and has been recognized as a devastating disease among cats for over half a century, yet despite marked efforts and many theories, the pathogenesis of FIP is still not fully understood [1,2]. Deficiencies in cell-mediated immunity promote the exuberant production of antibodies to feline coronavirus (FCoV), which results in antibody-dependent cytotoxicity and immune complex deposition [3,4,5]. Cats that resist FIPV are believed to mount a vigorous cell-mediated immune response and overcome the negative effects of FCoV antibodies [6]. Cell-mediated immunity is therefore believed to play an important role in controlling or eliminating the mutated virus. Cats in the terminal stages of FIP have severe depletion of the CD4+ and CD8+ T-lymphocytes necessary for mounting cell-mediated immunity [7,8]. Several cytokines have also been reported to play a role in FIP [9,10,11,12,13]. With immune dysregulation being a major component of the pathophysiology of FIP, treatment with an antiviral immunostimulant that triggers strong endogenous interferon production would be a rational approach.

While recent studies have reported the use of drugs like the nucleoside analog GS-441524 for the successful treatment of FIP, their availability in the US is limited, with only remdesivir (Veklury, Gilead USA) and nirmatrelvir and ritonavir (Paxlovid, Pfizer, USA) being FDA approved in humans [14,15,16]. It has been suggested that antiviral drugs can act synergistically with immunomodulatory treatments to improve patient outcome and survival in different viral diseases, such as influenza virus, hepatitis C virus, and human immunodeficiency virus (HIV) [17,18,19]. This synergism could be considered to improve long-term treatment outcomes for cats with FIP as well. 

Polyprenyl immunostimulant (PI) is believed to upregulate innate immunity via TLR2 and TLR6 [20] and is licensed by the U.S. Department of Agriculture for the reduction in the severity of signs of feline herpesvirus 1 (FHV-1) in cats over 8 weeks of age [21]. The product has also been evaluated in two open trials of cats with non-effusive FIP with some positive results [21,22]. The liposome Toll-like receptor 3/9 agonist complex (LTC) immune therapeutic has been studied in different veterinary species, including cats [23]. The effects of the LTC on the innate immune system has been well documented in healthy cats [23] and in follow-up studies showing effects against FHV-1 in a feline model [24,25]. Another study showed that a similar compound could be given safely by parenteral inoculation to cats with a positive effect on chronic rhinitis [26]. 

The objectives of this study were to determine the ability of the PI and LTC to stimulate the induction of type I (IFN-α, IFN-β) and type II (IFN-γ) interferon immune responses in vitro, to activate feline leukocytes to secrete factors capable of suppressing feline infectious peritonitis virus (FIPV) replication in vitro, and to induce evidence for immunomodulation when administered to healthy cats. The primary hypothesis was that both the PI and LTC would activate a variety of innate immune responses in cats that could be measured in vitro or in vivo. 

## 2. Materials and Methods

*Animals*: Purpose-bred cats for use in these experiments were originally purchased from Liberty Research (Waverly, New York, NY, USA). The use of the cats to provide peripheral blood mononuclear cells (PBMCs) for in vitro experiments and to assess two in vivo treatment protocols were approved by the Institutional Animal Care and Use Committee (Protocol # 170.063) of a local independent contract research facility. All young adult, mixed sex cats used in the study were group housed with multiple perches and other enrichment devices (ping pong balls; boxes to hide in) with ad libitum access to clean water and dry food. The cats had a complete physical examination performed by a study veterinarian prior to starting the study and then weekly (including body weight) for the duration of the study. All cats had complete blood cell count (CBC), serum biochemistry panel, and urinalysis performed prior to starting the study to assess for normalcy. The cats were assessed for vomiting, diarrhea, appetite, and attitude daily by facility employees for the duration of the study. After the completion of all experiments, the cats were adopted or returned to the colony in the research facility. 

In vitro *experiments with feline PBMC*: Feline PBMC (peripheral blood mononuclear cells) were extracted from whole blood of some of the healthy, untreated cats using Ficoll-Paque (Cytiva, Uppsala, Sweden) following manufacturer’s protocol. The PBMCs were incubated with LTCs and PI at indicated concentrations (1 µL per well, 0.3 µL per well, and 0.1 µL per well) in 24-well cell culture plates with 2 × 10^6^ cells per well in 1 mL of complete growth media (DMEM, 10%FBS, essential and nonessential amino acids, glutamax, and pen/strep). After 8 h, RNA was extracted using RNeasy kit (Qiagen, Hilden, Germany) according to manufacturer’s instructions, and RT-PCR was performed using GoTaq^®^ 1-step-PCR-system (Promega, Singapore) on QuantStudio 3 Real-Time PCR system. Primer and probe sequences used in the in vitro are shown in Table S1, and all primer and probe pairs were designed using IDT primer quest tool (IFN-α, IFN-γ, IL-1β, RPL30 housekeeping gene) and were verified by standard curve to have efficiency >95%. 

To detect the production of IFN-γ and IL-6, supernatants were collected from PBMC cultures with 1 × 10^6^ cells per well in 200 uL of complete media after 24 h. Cells were treated with increasing doses of PI or LTCs as indicated. Supernatants were assayed undiluted using a commercially available kits (Feline IFN-γ DuoSet ELISA and Feline specific IL-6 Duo Set ELISA R&D systems, Minneapolis, MN, USA) according to manufacturer’s instructions. 

*LTC protection from viral infection*: FIP virus (FIP79a) was propagated as previously described [27]. Optimal viral titer was determined using 50% viability. To assess the ability of LTC to trigger the release of cytokines capable of suppressing FIPV cytopathic effects on target cells (macrophages). The feline macrophage cell line (fcwf-4, ATCC American Tissue Culture System. Manassas, VA, USA) was grown in cell culture with MEM containing 10% FBS and was used for FIPV infection and cytopathicity studies.

After the fcwf-4 cells were confluent, they were incubated with supernatant from LTC-treated feline PBMC cultures (or untreated PBMC supernatant for control); then, FIPV was added at an MOI of 3 and incubated at 37 °C and 5% CO_2_ for another 4 h. After 4 h, the FIPV supernatant was washed away, and the Fcwf cells were incubated for another 72 h in complete growth medium containing FBS. At 72 h post infection, non-adherent dead cells were washed away, and each well was imaged using Olympus CKX41 inverted brightfield microscope. Live cells were counted using ImageJ [28].

In vivo *PI and LTC treatment study design*: In the in vivo treatment study, 12 young adult cats (6 males; 6 females) were randomly housed in two groups (6 cats in the PI group and 6 cats in the LTC group, each in a 12′ × 14′ room). The PI product was purchased from the manufacturer. The LTC was prepared as described previously [23]. Each cat that was randomized to the PI group was administered PI at 3 mg/kg orally once a day for 14 days, as recommended by the manufacturer. Cats that were greater than 5 kg were administered a maximum dose of 15 mg. All cats were monitored for 30 min after each dose of PI for vomiting. 

For treatment with LTC, a dose of 1 mL was selected for evaluation based on prior experience with LTC administered topically in cats [24,25] and in dogs as a cancer vaccine adjuvant [29]. All cats in the LTC group were administered LTC 1 mL s.c. twice weekly for 2 weeks. Temperatures were recorded before LTC or PI administration and at 12 and 24 h after administration. For cats treated with LTC, injection sites were examined for skin reaction and pain prior to each LTC injection and at 12 h and 24 h after each injection. All cats had complete blood cell count (CBC), serum biochemistry panel, and urinalysis performed at the end of the study to assess for side effects.

In vivo *PI and LTC treatment study immune assays*. Blood was collected from all 12 cats prior to the administration of the PI or LTCs and then on days 1, 3, 7, and 14 for serum separation prior to assay for 19 different cytokines and chemokines (sFas, Flt-3 ligand, GM-CSF, IFN-γ, IL-1β, IL-2, IL-4, IL-6, IL-8, IL-12 (p40), IL-13, IL-18, KC, MCP-1, PDGF-BB, RANTES, SCF, SDF-1, TNF-α) by the use of a commercially available kit (MILLIPLEX MAP Feline Cytokine/Chemokine Magnetic Bead Panel Premixed 19 Plex, FCYTOMAG-20K-PMX—Immunology Multiplex Assay, Millipore Sigma), and assays were performed according to the manufacturer’s instructions. Whole blood in EDTA was subjected to Ficoll density gradient separation to produce PBMCs (see below). The PBMCs were stimulated in vitro and used in quantitative reverse-transcriptase PCR assays (qRT-PCRs) to amplify the mRNA of select cytokine genes (IFN-α, IFN-γ, IFN-β, IL-1β, IL-6, and TNF-α) and Glyceraldehyde-3-phosphate dehydrogenase (GAPDH, a housekeeping gene) using previously published techniques [23,30]. 

*Statistical methods*: In the feline PBMC experiments, comparisons amongst control wells and the 3 concentrations of PI and LTCs were compared using ANOVA, followed by the Tukey multiple means post-test (Prism9 software; GraphPad, La Jolla, CA, USA). Differences in the extent of cytotoxicity induced by FIPV were compared between treatment groups using a non-parametric *t* test. In the in vivo PI versus LTC cat treatment study, the results of assays over time were compared between the groups using repeated measures ANOVA and between groups at individual time points by two tailed Student’s t test. For all analyses, statistical significance was set at *p* < 0.05.

## 3. Results

**Figure 1:**
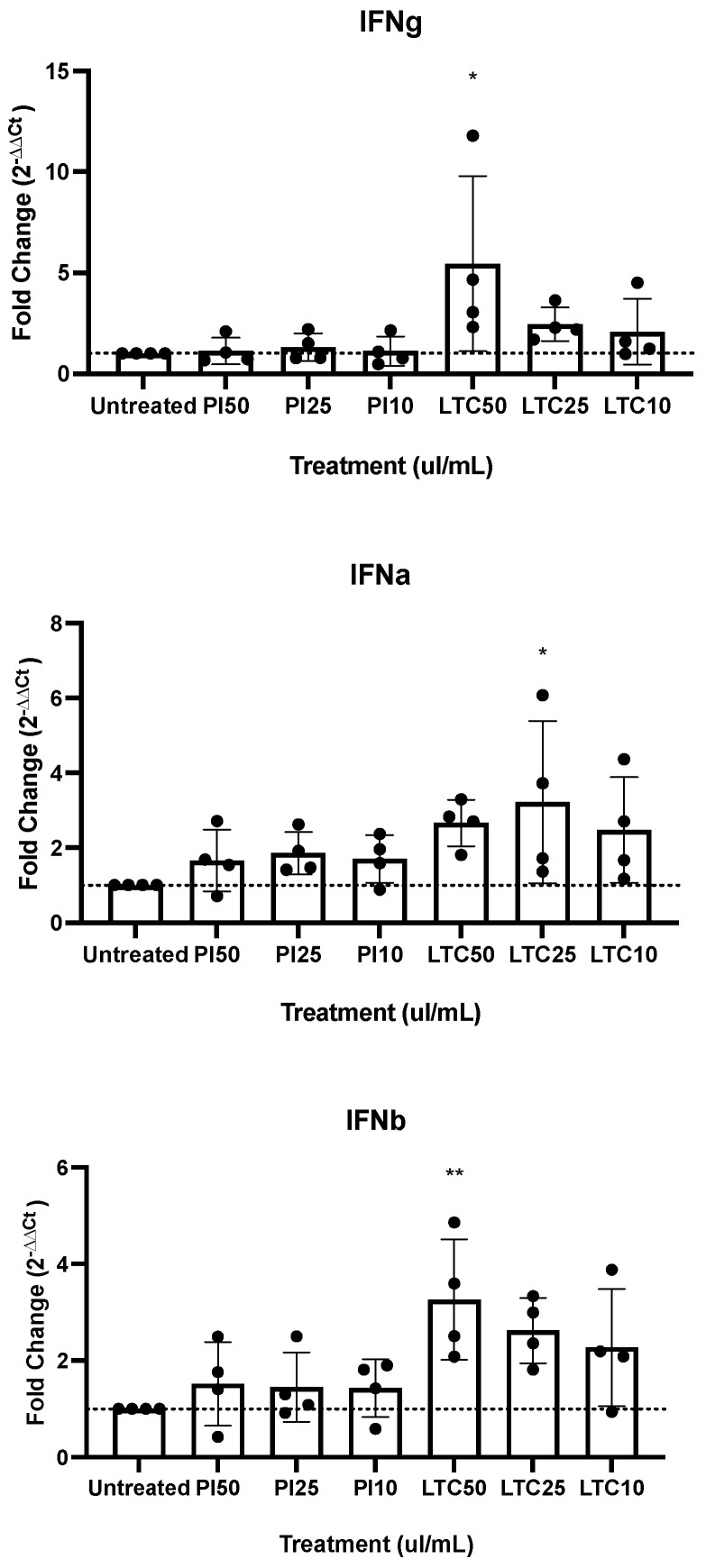
In vitro activation of expression of IFN-γ, IFN-α, and IFN-β measured by RT-PCR of specific mRNA after an 8-h incubation of feline PBMCs with three different concentrations of PI (PP), and LTC concentrations are shown in a decreasing scale of 50 µL/mL (1mL media per well), 25 µL/mL, and 10 µL/mL. LTC at concentration 50 µL/mL induced significant expression of interferon IFN-γ (*p* = 0.0152). LTC at concentration 25 µL/mL induced significant expression of IFN-α (*p* = 0.0441), and IFN-β had induced significant expression by LTC at concentration of 50 µL/mL (*p* = 0.0060). Significance * *p* < 0.05 and ** *p* < 0.01.

In vitro *experiments with feline PBMC*: The in vitro studies were set up to assess and compare the immunological activities of two compounds for cats. The first study used in vitro cultures of PBMC from healthy cats to assess the induction of expression of genes encoding six key antiviral cytokines (IFN-a, IFN-g, INF-b, TNF-a, IL-1b, and IL-12). When compared to untreated PBMC, the significant expression of IFN-α, IFN-β, or IFN-γ was not induced by PI at any of the three concentrations studied (Figure 1). In contrast, the LTC induced significant increases in all three cytokines, with variation amongst different concentrations (Figure 1). 

**Figure 2:**
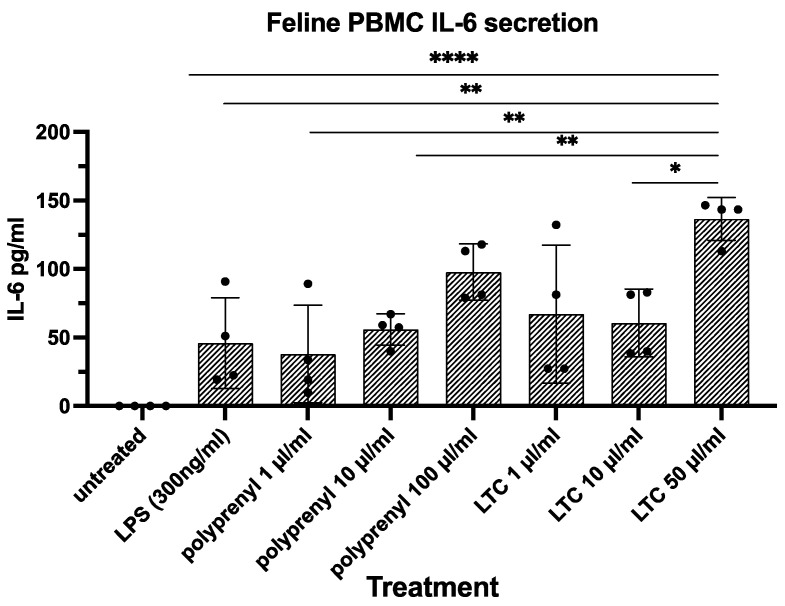
IL-6 secretion by feline PBMCs: polyprenyl vs. LTC. Statistical differences were obtained using a one-way ANOVA with Tukey’s multiple comparisons test with ****, *p* ≤ 0.001; **, *p* ≤ 0.01; and *, *p* ≤ 0.05.

ELISA assays for feline proinflammatory cytokines IL-6 and IFN-γ were used to determine their release by immune-activated PBMC cultures assessing the impact of LTC and PI treatment on secretion by feline PBMC. The proinflammatory cytokines IL-6 and INF-γ were measured in vitro from feline PMBCs treated with various concentrations of either PI or LTC. LTC was generally a stronger immune stimulant than PI at high concentrations (Figure 2) with higher concentrations of this formulation of LTC mediating the highest release of IL-6. 

**Figure 3:**
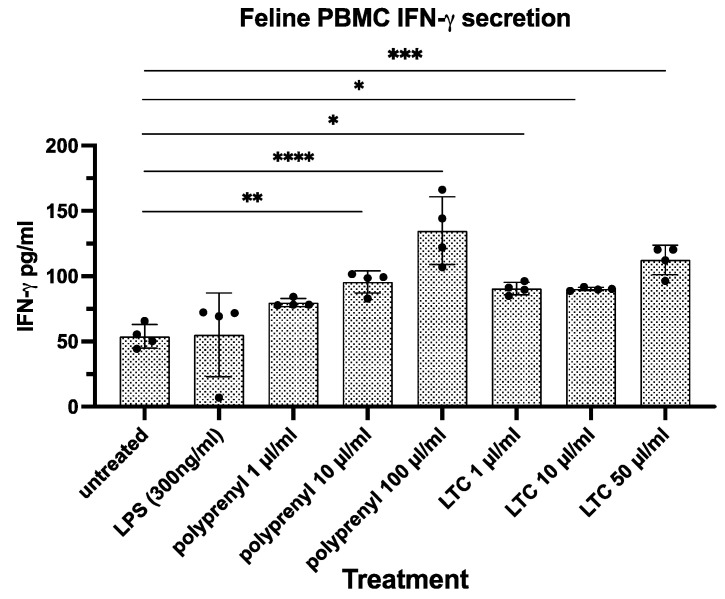
IFN-γ secretion by feline PBMCs: polyprenyl vs. LTC. Statistical differences were obtained using a one-way ANOVA with Tukey’s multiple comparisons test with ****, *p* ≤ 0.001; ***, *p* ≤ 0.005; **, *p* ≤ 0.01; and *, *p* ≤ 0.05.

Relative to untreated PBMC, IFN-γ was significantly increased in PI-treated PBMCs at 20 and 200 µg/mL and in cells treated with all concentrations of LTCs (1, 10, and 50 µg/mL) (Figure 3). 

**Figure 4:**
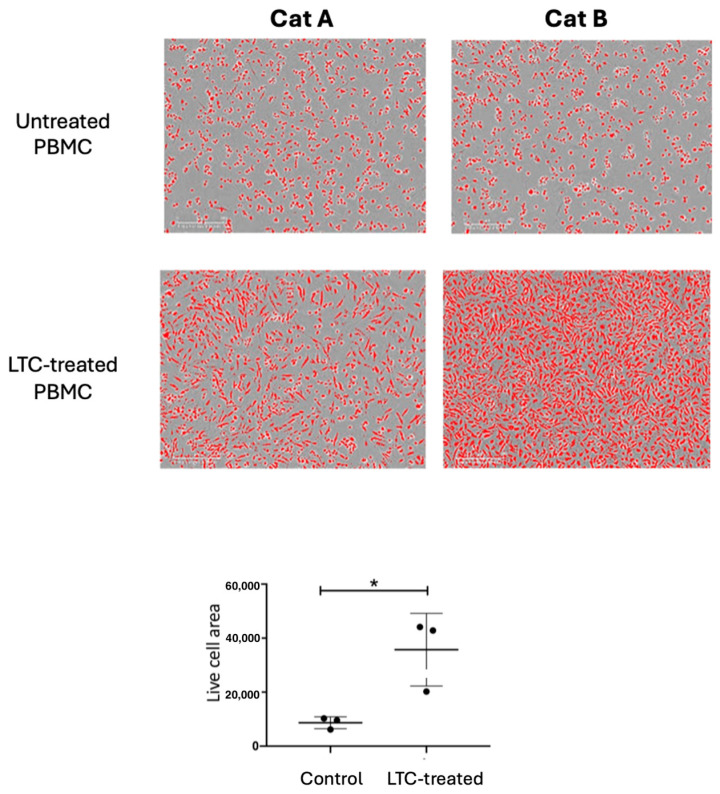
LTC activation of feline leukocytes prevents cytopathic effects imposed by FIPV. Untreated cells (A), or cells treated with LTC overnight (B) were inoculated with virulent FIPVs to assess cytopathic effects and cell survival (live cells appear as red dots in these images). Cell survival data are summarized in C, illustrating significant protection from FIPV-induced killing by factors from LTC-activated leukocytes. Significance defined as * *p* ≤ 0.05 using non-parametric Mann–Whitney *t*-test.

*LTC protection from viral infection*: Given that LTC was shown above to activate production and secretion of key antiviral cytokines (whereas PI did not), we next assessed the ability of LTC to stimulate the secretion of antiviral cytokines capable of suppressing FIP replication and cytopathicity. These studies used feline PBMCs as the source of antiviral cytokines, given that therapeutic administration of LTCs would involve the activation of immune cells in lymph nodes and blood. As the readout for antiviral (anti-FIP) activity, the feline tumor macrophage cell line fcwf-4, which has been used previously for FIP replication and treatment studies [27], was used. There was significant protection from FIPV-induced cytopathic effects when fcwf-4 cells were treated with conditioned medium from LTC-activated leukocytes (Figure 4). 

*Evaluation of systemic immune responses to treatment with PI or LTCs in healthy cats*: Based on the preceding studies, which demonstrated the ability of LTC to potently activate innate immune responses in cat PBMC, we next determined whether the parenteral administration of LTCs (s.c. injection) would elicit detectable systemic immune responses, using serum cytokine analysis and cytokine RT-PCR for PBMC isolated directly from blood of treated cats. Healthy, purpose-bred cats were treated with PI by oral dosage and/or treated by s.c. injection with LTC and serum cytokine responses were monitored. The dose and route of administered PI were based on the manufacturer’s recommendations [22]. For LTC administration, the dose and route of administration were selected based on prior studies in cats with LTC as a topical immune stimulant [24,25] and in dogs as a cancer vaccine adjuvant for s.c.-administered vaccines [29].

Study animals were also monitored for adverse effects due to PI or LTC administration. Cats treated orally with PI did not exhibit any adverse clinical signs. One cat in the LTC group experienced pain for 24 h at the local site after one injection. No other side effects were noticed in any of the cats in the LTC group or PI group. No changes in CBC, serum biochemistry panel, or urinalysis were noted in the cats in the PI group at the end of the study when compared to prior to the study. Two cats in the LTC group had mild increases (one cat 1.1× and one cat 3.3× the upper reference interval) in alanine aminotransferase and aspartate aminotransferase activities. These values normalized in one cat but remained 2.7× the upper reference interval in the other cat two weeks after the last dose of LTC. 

For the in vivo evaluation of LTC and PI immune responses, we first assessed concentrations of circulating cytokines, using a feline cytokine multiplex panel of 19 cytokines. For all cats, regardless of day or group, sFas, GM-CSF, IFN-γ, IL-1β, IL-2, IL-4, IL-6, IL-8, IL-13, IL-18, KC, MCP-1, SCF, and TNF-α were below the limit of detection. The assay was able to detect the measurable concentrations of the following cytokines in either pre- or post-treatment blood samples from animals in this study (Flt-3 ligand, SDF-1, IL-12 (p40), and PDFG-BB). Of the cytokines that could be measured, we observed only significant changes in concentrations of the chemokine RANTES (*p* < 0.02) in cats treated with LTCs and not in cats treated with PI. 

**Figure 5:**
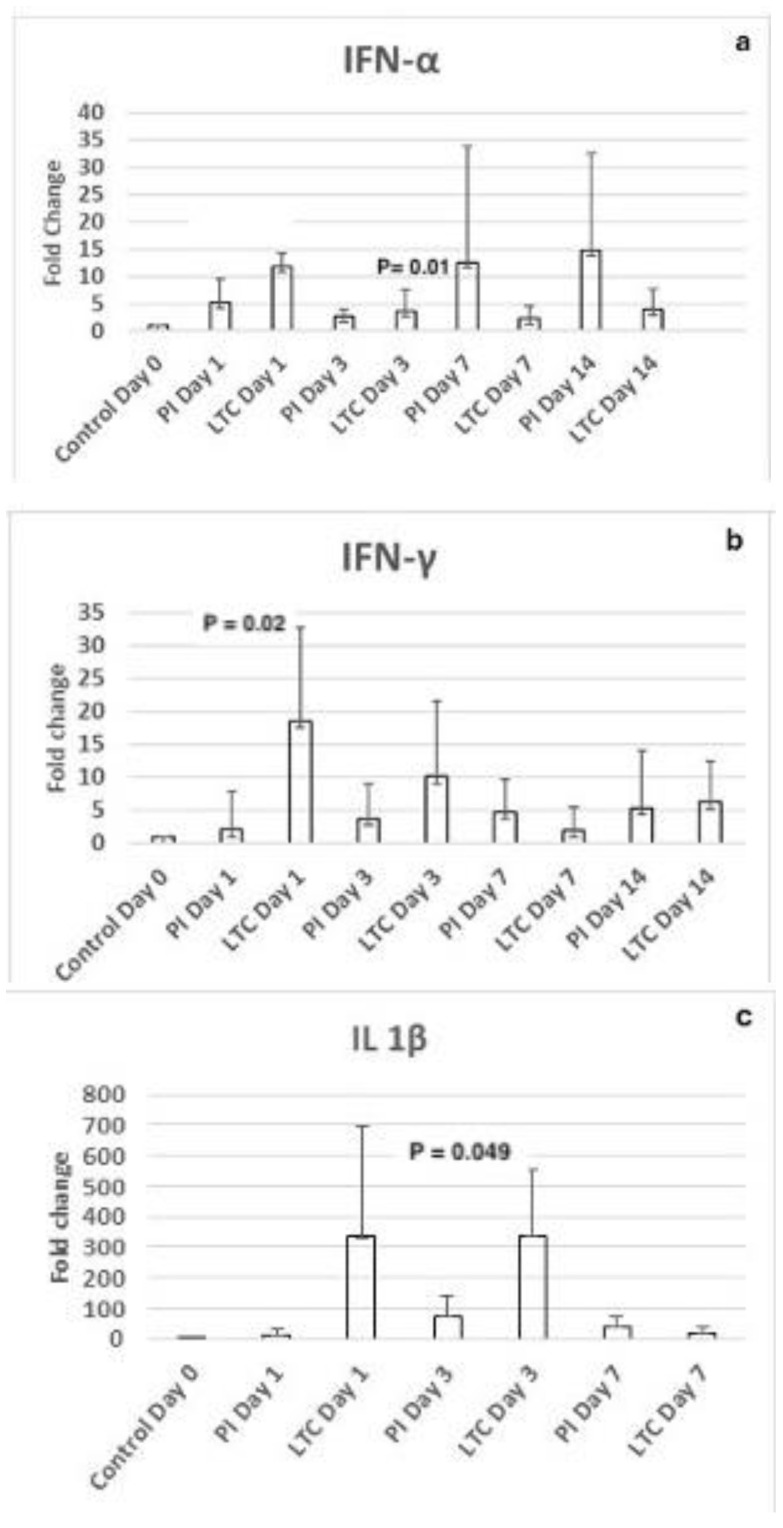
Fold change increases in IFN-α (**a**), IFN-γ (**b**), and IL-1β (**c**) mRNA at Days 1, 3, 7, and 14 in cats administered the LTC or PI protocols. When results were significantly different between groups on individual days, the *p* value is posted above the values.

Finally, the impact of LTC and PI treatment on the immune response by circulating leukocytes was also evaluated by using targeted RT-PCR for key antiviral cytokines and PBMCs from treated animals, which were measured directly (i.e., without culture). Increases in cytokine gene expression above baseline values (fold change; 2^−ΔΔCT^) were noted at all time points for IFN-α, IFN-γ, and IL-1β (but not for IL-6 or TNF-α), with several statistical differences noted between the groups. When compared to PI treatment, there was significantly higher mRNA expression for both INF-a and IFN-γ at Day 1 post-treatment and for IL-1b at Day 3 in LTC-treated cats (Figure 5). 

## 4. Discussion

The primary objective of this study was to compare the immunological and antiviral activities of two innate immune stimulants (PI and LTC) using in vitro studies with cat PBMCs and cat macrophages as well as in healthy cats being treated with PI and LTCs. 

In the in vitro study, the LTC doses studied induced significant production of IFN-α, IFN-β, and IFN-γ by stimulated feline leukocytes, whereas PI failed to induce these interferons at any dose. These findings are consistent with prior reports from our group, wherein LTC was found to activate local immune responses and the in vitro activation of PBMCs in cats [23]. These findings suggest that activating the TLR 3 and 9 pathways is an effective approach for generating broad interferon production in cats. Moreover, LTC seems to be a stronger immune stimulant for IL-6 release than PI at high concentrations, and a high concentration of this formulation of LTC does not seem to be as toxic in terms of IL-6 production, or it is also possible that the cells released IL-6 before dying. Interleukin-6 plays a central role in host defense due to its wide range of immune and hematopoietic activities and its potent ability to induce the acute phase response, which is an important response in cats with FIP. IL-6 is also known to have antiviral effects [31]. There was a significant increase in IFN-γ secretion in PBMC treated with 10.0 µL/mL LTC; however, higher doses of LTC seem to be toxic to cat PBMCs in vitro and lessen IFN-γ secretion. 

In addition, treatment of PBMCs with LTCs stimulated the production of cytokines that significantly suppressed the cytopathic effects of FIP in feline macrophage cultures. Thus, these studies revealed the potent innate immune-activating and antiviral activities of LTCs, compared to PI, for therapeutic immune stimulation in cats. This in vitro experiment also showed that there was significant protection from FIPV-induced cytopathic effects when fcwf-4 cells were treated with conditioned medium from LTC-activated leukocytes, showing that the cytokines triggered by LTC activation are able to suppress FIPV replication in permissive macrophages, a key target cell for FIP pathogenesis. Thus, treatment of PBMCs with LTCs stimulated the production of cytokines that significantly suppressed the cytopathic effects of FIP in feline macrophage cultures. Together, these studies revealed the potent innate immune-activating and antiviral activities of LTCs, compared to PP, for therapeutic immune stimulation in cats. 

When healthy cats were treated with LTCs and PI, neither protocol induced significant side effects, and the increases in liver enzyme activities induced by the LTC protocol were self-limited. The biological activity of PI and LTC were also assessed in vivo in healthy cats. The primary outcome measure for these studies was cytokine secretion, as assessed by feline multiplex assay (19 cytokines). When serum samples obtained after PI or LTC administrations were assessed, we found that LTC administration induced the significant upregulation of RANTES secretion. RANTES attracts immune cells from the peripheral blood to sites of inflammation and plays an important role in homing and migration of effector and memory T cells during acute infections. RANTES has been associated with decreased susceptibility to the human immunodeficiency virus [32]. Hence, RANTES may increase effective cell-mediated immunity that is often reduced in FIP-prone cats. Other cytokines were either not detected (sFas, GM-CSF, IFN-γ, IL-1β, IL-2, IL-4, IL-6, IL-8, IL-13, IL-18, KC, MCP-1, SCF, TNF-α) or were not increased by PI or LTC administration. The failure to detect greater in vivo immune activation using the cytokine assay as a readout may reflect in part the relative insensitivity of circulating cytokine measurement for systemic immune activation. In addition, while the dose of PI administered was the dose recommended by the company marketing PI, the dose of LTC administered was not optimized for s.c. administration. Our prior studies have found, for example, that the route of LTC administration greatly affects the degree of immune activation that is achieved at the actual dose administered [29]. It is also possible that PI administration may induce local immune activation in the gut, while LTC may induce local immune activation in s.c. tissues and draining lymph nodes, and that neither of these routes would be sufficient to induce changes in concentrations of circulating cytokines. 

Both the PI and LTC protocols in healthy cats induced fold increases over baseline for IFN-α, IFN-γ, and IL-1β but not IL-6 or TNF-α. IL-1β had the greatest fold increases and is known to amplify IFN-α-induced antiviral responses [33]. Significant differences between the protocols existed with responses being greater for the LTC protocol on either Day 1 (IFN-α, IFN-γ) or Day 3 (IL-1β) of the study. Whether there is biological significance of the detection of greater or earlier responses for these three cytokines is unknown, and the findings could merely reflect the route of administration with the SQ route inducing changes more quickly. 

It has been suggested that antiviral drugs can act synergistically with immunomodulatory treatments to improve patient outcome and survival in different viral diseases, such as influenza virus, hepatitis C virus, and human immunodeficiency virus (HIV) [17,18,19]. Overall, the results of the in vivo and in vitro experiments reported herein support the LTC protocol described to have antiviral properties and should be considered for use in combination with antiviral drugs. This synergism could improve long-term treatment outcomes for cats with FIP as well. In addition, the induction of antiviral innate immune responses could be very beneficial for the management of a variety of feline infectious diseases, not only FIP, but also for FHV-1, feline caliciviruses, and *Mycobacteria* spp. that still lack effective treatments or have treatments that are expensive, difficult to administer, or are toxic. Considering the results of our in vitro study, it is likely that LTCs can be effective in the treatment of FIP and other infectious diseases, and additional studies are warranted to assess the immune-stimulatory properties of LTCs and to rigorously evaluate their general antiviral properties in vivo, as well as their potential effectiveness as a treatment for FIP. 

In contrast to the potential benefits of immune stimulation, it is also possible to potentiate disease. Several cytokines have previously been reported to play a role in FIP. An older study reported that the mRNA levels of IL-2, IL-4, IL-10, IL-12, and IFN-γ were markedly reduced during the development of FIP in kittens [9]. A recent study measured the mRNA levels of IL-1β, IL-6, and TNF-α in liver and heart of cats with FIP and showed that both hepatocytes and cardiomyocytes are sources of inflammatory cytokines in FIP, with hepatic IL-12:IL-10 balance skewed towards IL-12 in cats with FIP [13]. In another study, IL-1β, IL-6, IL-12, IL-18, TNF-alpha, macrophage-inhibitory protein (MIP)-1 alpha, and RANTES showed moderate upregulation in cats with neurological FIP and very high upregulation in cats with non-neurological FIP [10]. In the same study, the transcription of IFN-γ appeared upregulated in cats with systemic FIP and slightly downregulated in neurological FIP; however, most cytokines in this study had very high variance in non-neurological FIP, but less variability was noted in neurological FIP [10]. Various proinflammatory cytokines and interferon-related genes such as MX1, viperin, CXCL10, CCL8, RANTES, KC, MCP1, IL8, GM-CSF, and IFN-γ were found in FCoV-positive cats [12]. Cats with FIP have also been reported to have higher IL-6 expression, and IL-6 is likely involved in the development of immune-complex-mediated vasculitis and in FIP pathogenesis [11]. RANTES expression was previously reported to be high in cats with FIP compared to healthy cats [10], suggesting it as a possible target for immunotherapy of cats with FIP. However, dysregulated excessive and persistent synthesis of IL-6 has a pathological effect on acute systemic inflammatory response syndrome and chronic immune-mediated diseases, and therefore, immunotherapy, which might be increasing RANTES and IL-6, might not be as beneficial long term for these cats. There is some clinical information available for the two immune stimulants described here that suggest that a clinical benefit for their use with viral infections is more likely than the potentiation of disease with FIP and FHV-1 [22,24,25,34,35], and additional clinical studies in cats with acute or chronic infectious diseases are warranted.

Other than the increased expression of RANTES in the LTC group, neither protocol used in the in vivo experiment induced many changes in the production of the cytokines and chemokines measured in the commercial kit used with healthy cat serum. This commercial kit has shown low values for many analytes in other studies using serum or plasma of cats [36,37]. In one of these studies, over half of the samples were below the lower limit of quantification for both serum and plasma for nine analytes (FAS, GM-CSF, IFN-γ, IL-1β, IL-2, IL-6, CXCL-1, PDGF-BB, and TNF-α), and additional analytes were below the level of detection in plasma samples only [36]. These results suggest that this kit may not be optimal for attempting to determine changes in cats with minimal immune simulation or inflammation. Other assays, like the PCR assays described here, or bulk RNA sequencing may be more sensitive for use in healthy cat studies [38]. There were several limitations to the study. First, the numbers of study animals were small and reflect the pilot nature of these investigations. Timing could have played a role in the measurements as the cytokines were only measured at one time point, and measurements would ideally be taken at various post-treatment times to find out the optimal time of secretion before cytotoxicity predominates, which was not performed in this study as these were single time points. Another limitation is that these are in vitro and healthy cat studies that may not fully reflect the effectiveness in cats with FIP, and an in vivo study using immune stimulants such as LTCs and PI should be performed in cats with FIP as an adjunct treatment to antiviral therapy to further evaluate the effectiveness of these immune stimulants in the treatment of FIP.

## 5. Conclusions

Both protocols studied herein showed evidence for immune modulation that could be of potential benefit to the management of viral infections. While differences were noted between the LTC (TRL3/TRL9) and PI protocols (TLR2/TLR6), care should be taken in concluding that one is better than the other since the routes of administration were different, and there has not been an optimized dose determined for either agent. The studies reported here indicate that the LTC is a potent innate immune agonist in cats, capable of eliciting a high expression of key antiviral genes, including IFN-a, IFN-b, IFN-g, and IL-1b in feline PBMC. In addition, LTCs generated significant antiviral activity against FIP replication in feline macrophages. Therefore, we conclude that the LTC was as effective as an innate immune agonist for activating feline antiviral responses both in vitro and in vivo and should therefore be considered a promising new addition to treatment options for FIP and other chronic viral pathogens of cats, and additional studies are warranted to assess the immune-stimulatory properties of LTCs and to rigorously evaluate their antiviral properties in vivo, as well as their potential effectiveness as treatments for FIP.

## Data Availability

The authors confirm that the data supporting the findings of this study are available within this article and/or it’s supplementary materials.

## Supplementary Materials

The following supporting information can be downloaded at: https://www.mdpi.com/article/10.3390/pathogens13070602/s1; Table S1: Primer and probe sequences for quantitative PCR assays used in the PBMC experiments.
