# Supplementary material for: Comparison of Antiviral Immune Responses in Healthy Cats Induced by Two Immune Therapeutics
Source: Pathogens. 2024 Jul 22;13(7):602. doi: 10.3390/pathogens13070602 (PMC11280254; doi:10.3390/pathogens13070602)
Supplement: Supplementary file 1 [file pathogens-13-00602-s001.zip › pathogens-3024340-supplementary.pdf]

Supplementary Materials

Table S1: Primer and probe sequences for quantitative PCR assays used in the PBMC experiments

Feline IFNg<br>NM_001009873.1	Fwd: TGACTTCCTCAAGCTGATTC
	Rev: TCCTCAGGTTAGATCTTGGT
	Probe: /56-FAM/CTGCAGGTC/ZEN/CAGCGCAAAGCAAT/3IABkFQ/
Feline IFNa1<br>NM_001245020	Fwd: CTGTCAGAAGGACAGAAGTG
	Rev: GTGCAGAAGAAGTGGAAGAT
	Probe: /56-<br>FAM/TGTTTGGTG/ZEN/GAGACCAGTCCCACAA/3IABkFQ/
Feline IFNb1<br>NM_001009297.1	Fwd: CACTGTTGAGAACCTCCTTG
	Rev: GGTTCAGAAGGGTCGTATTG
	Probe: /56-<br>FAM/TGGCAGAAG/ZEN/GAACACCTGGAAACGA/3IABkFQ/
Feline RPL30<br>XM_006943321.4	Fwd: AAGCGAAACTGGTCATCC
	Rev: ATTGCCGCTGTAGTGATG
	Probe: /56-<br>FAM/AACAACTGC/ZEN/CCAGCCTTGAGGAAGT/3IABkFQ/
